# Data of microstructure and mechanical properties of carbon foams derived from sucrose/polyacrylamide hydrogel

**DOI:** 10.1016/j.dib.2016.02.022

**Published:** 2016-02-15

**Authors:** Yao Yao, Fei Chen, Xi Chen, Qiang Shen, Lianmeng Zhang

**Affiliations:** State Key Lab of Advanced Technology for Materials Synthesis and Processing, Wuhan University of Technology, Wuhan 430070, China

**Keywords:** Carbon foams, Material design, Mechanical properties

## Abstract

An easy method that combined gel casting and physical foaming was used to fabricate modified carbon foams. The design of carbon foams from sucrose/polyacrylamide hydrogel is a new concept for controlling the microstructure and improving the compressive properties of carbon foams. This article provides the micrographs obtained from optical and scanning electron microscope for foaming solution and carbon foams. Weight loss data used to construct the thermo-gravimetric curves are included. Load–displacement data constructing the stress–strain curves and the derived compressive properties are also included.

## Specifications table

**Table:** 

Subject area	Materials Science and Engineering.
More specific subject area	Material Design/Characterization.
Type of data	Figures and images (optical and scanning electron micrographs), Tables and graph (thermo-gravimetric curves, compressive stress–strain curves).
How data was acquired	Following techniques were used for acquiring data: optical microscope, scanning electron microscope, thermo-gravimetric analyzer, universal testing machine.
Data format	Images, raw data in csv format, analyzed and plotted in tables and graphs.
Experimental factors	Sucrose/polyacrylamide hydrogel was chosen in this new method combining gel casting and physical foaming. 2 wt% anionic surfactant sodium dodecyl sulfate (SDS) was added to modified the foaming process.
Experimental features	The carbon precursor was thermal polymerized in an inert atmosphere at 350 °C for 5 h. Compressive properties such as the compressive strength, plateau stress and modulus are derived from data.
Data source location	Wuhan University of Technology, Wuhan, China.
Data accessibility	Data is provided with this article.

## Value of the data

● From the present data, carbon foams with uniform cell structure can be prepared from sucrose/polyacrylamide hydrogel by combining gel-casting and physical foaming methods.
● The provided data are suitable for comparing the compressive strength of carbon foams with the similar density prepared by other carbon precursor and method to make selection of the best raw materials and optimum foaming method.
● Data obtained from optical and scanning electron microscope can be used to control the microstructure of carbon foams, which aims to predict the properties of carbon foams.
● The present data can be used as input parameter in the further studies on finite element analysis of the mechanical behavior of carbon foams.

## 1 Data

Original optical micrographs of the foaming solution was observed by the digital optical microscope (DOM; LM, DM-2500M, Germany). The data is presented to illustrate the modification of anionic surfactant sodium dodecyl sulfate in the foaming process.

Micrographs of carbon foams was obtained by field emission scanning electron microscopy (FESEM; JEOL, JSM-7500F, Japan) at an accelerating voltage of 20 kV. The data is presented to show the microstructure of carbon foams.

The weight loss data of the carbon precursor prepared from sucrose/polyacrylamide hydrogel was carried out in both air and argon atmosphere up to 900 °C using a thermo-gravimetric analyzer (NETZSCH, STA449F3, Germany). Heating rate used was 5 °C/min. The data can be processed to convert to TGA curves of the carbon precursor in argon and air atmosphere.

Bulk density ($\rho_{b}$) of carbon foams was obtained by dividing the mass by the volume of samples of known dimensions. Skeletal density ($\rho_{s}$) was measured by the pycnometer test method [1]. Samples were crushed into powder to avoid any erroneous results related to possibly closed porosity. The skeletal densities of all samples are 2.00±0.01 g/cm^3^ which is the typical density of amorphous carbon materials [2]. The porosity (*φ*) was calculated according to the following equation [2]:

$$\varphi =1-\frac{\rho_{b}}{\rho_{s}}$$

Load–displacement data obtained for all specimen׳s tested under compression is presented. Compression testing was conducted using a universal testing machine according to ASTM Standard C365/C365M-05. Bluehill 2.0 software was used to acquire force–displacement data from the machine. The data can be processed to convert to stress–strain curves and calculate various compressive properties in carbon foams.

## 2 Experimental design, materials and methods

### 2.1 Experimental design

The carbon precursor of carbon foams was manufactured from the sucrose/polyacrylamide hydrogel using gel casting and physical foaming technique. Before the catalytic polymerization and crosslink of polyacrylamide hydrogel, anionic surfactant sodium dodecyl sulfate was added to enhance the foaming ability and stability under the physical high-speed stirring.

The carbon precursor was thermal polymerized in an inert atmosphere in a tubular furnace at 350 °C for 5 h. In order to prevent the deformation and cracks during the heat treatment, the carbon precursor of carbon foams was moisturized and dried at room temperature for 24 h. The pre-heated samples were carbonized in the same atmosphere at 1000 °C for 2 h. Ultra-high purity argon gas (99.999%) maintaining all heat treatment of foams was used as the inert atmosphere. The heating rate used for all heat treatment was 2 °C/min.

### 2.2 Microstructure

**Fig. 1:**
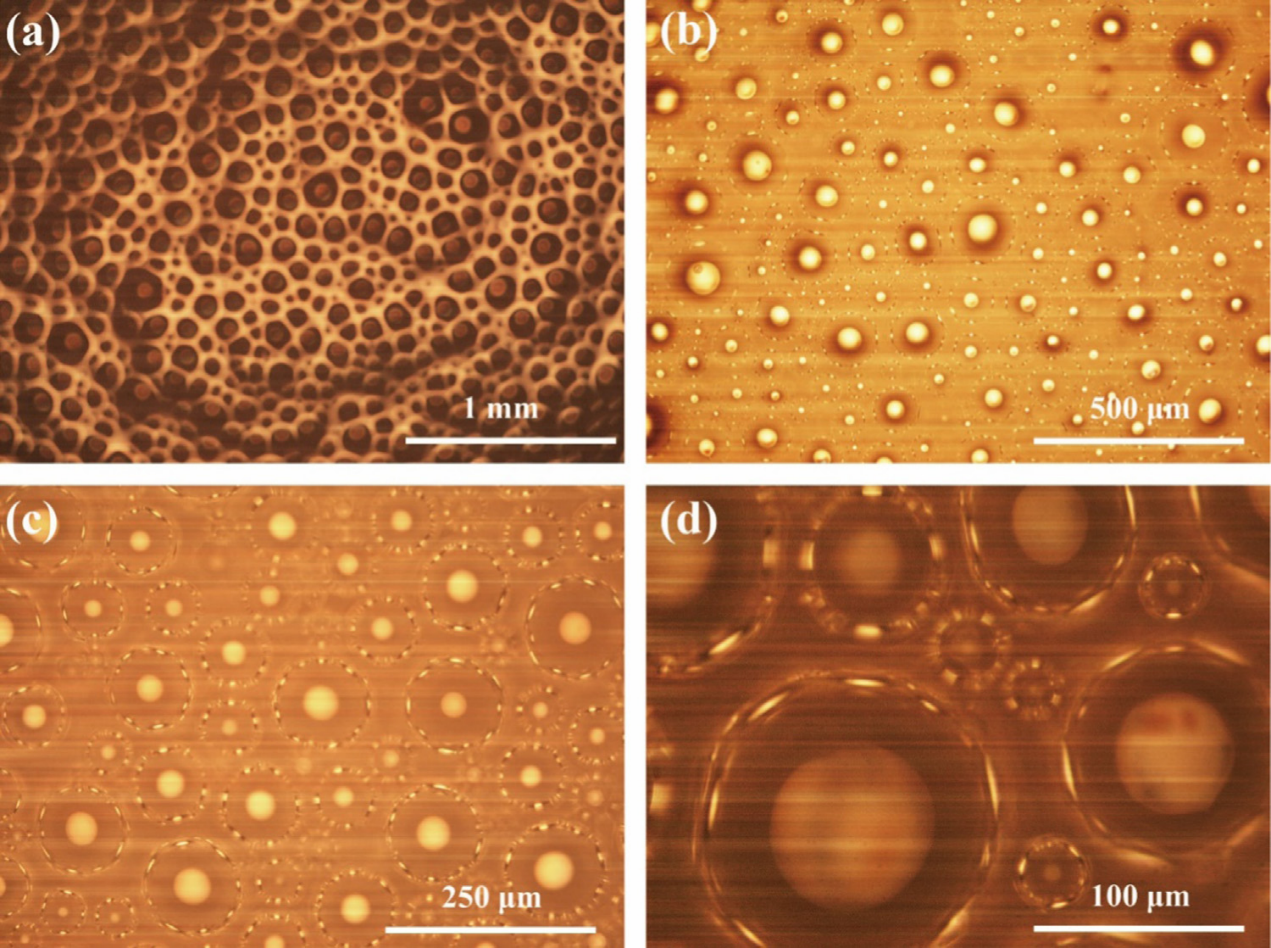
Optical micrographs of the foaming solution at different magnifications.

Fig. 1(a) shows that these gas bubbles stably exist in the foaming solution with the uniform size at the end of the physical foaming process. High porosity present in the foaming solution can be observed in this image. Fig. 1(b), (c) and (d) show the sodium dodecyl sulfate tends to adsorb at air–water interfaces [3].

**Fig. 2:**
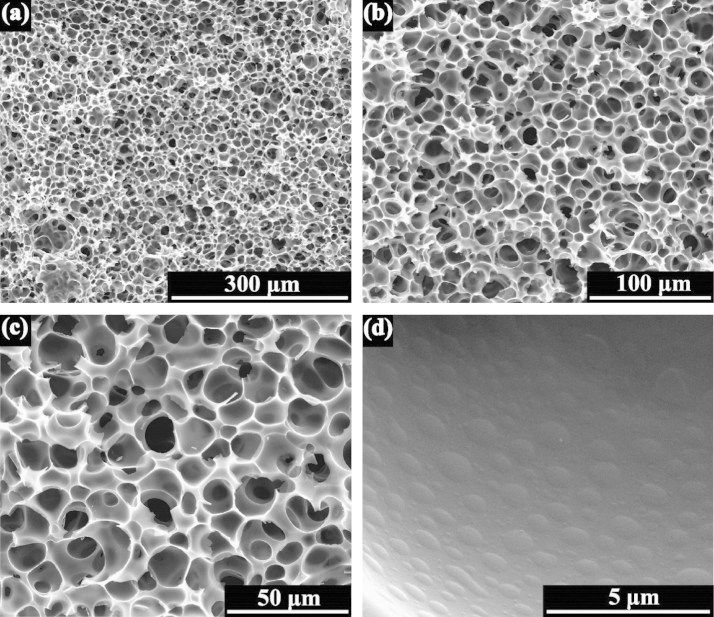
(a), (b), (c) SEM images of carbon foams at different magnifications, (d) the cell wall structure of carbon foams.

Fig. 2(a), (b), (c) show the microstructure of carbon foams. The carbon foams have an interconnected cellular structure with spherical cells. The open cells being interconnected through oval pores (also called windows [4]) are evidenced, being speared with each other by intact junctions and cell walls. The carbon foams have a good microstructure in which the junctions in the skeleton of carbon foams are intact and the ligaments are smooth. Little micro cracks can be observed from the images. As shown in Fig. 2(d), many regular elliptic pits on the cell wall are exhibited.

### 2.3 Thermogravimetric characterization

**Fig. 3:**
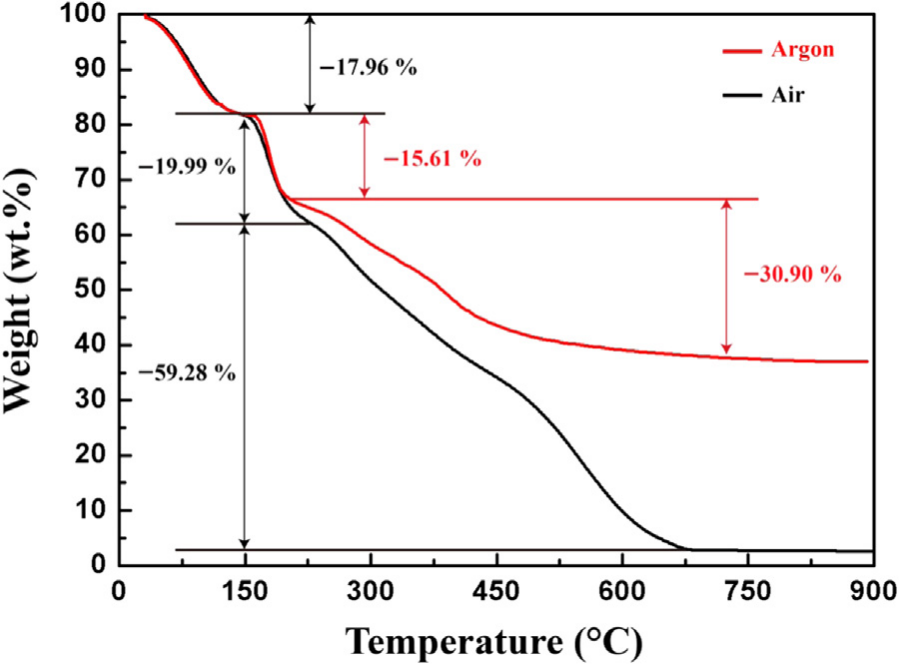
Thermo-gravimetric curves of the carbon precursor in argon and air atmosphere.

After acquiring the weight loss data using the thermo-gravimetric analyzer, thermo-gravimetric curves were plotted as shown in Fig. 3. The raw data obtained during the test can be found in files with nomenclature to RawData_air.csv, where “air” refers to the atmosphere.

The carbon precursor shows the similar thermal decomposition pattern in both air and argon atmosphere in the temperature range of 0–200 °C [5]. The dehydration continues along with the oxidation of carbon at the temperatures above 200 °C in air atmosphere and leaves only 2.77 wt% carbon yield at 900 °C. On the other hand, nearly 35.53 wt% carbon yield is retained at 900 °C in argon atmosphere.

### 2.4 Compressive characterization

A constant displacement rate of 0.5 mm/min was applied for all specimens. Specimens were tested with the dimensions 6×6×3 mm^3^. Five specimens were tested and compressive properties are calculated and presented in Table 1. All of these specimens were prepared at the same contents of sucrose (50 wt%) and SDS (2 wt%). The raw data obtained during the test can be found in files with nomenclature to Specimen_RawData_1.csv, where “1” refers to specimen number.

**Fig. 4:**
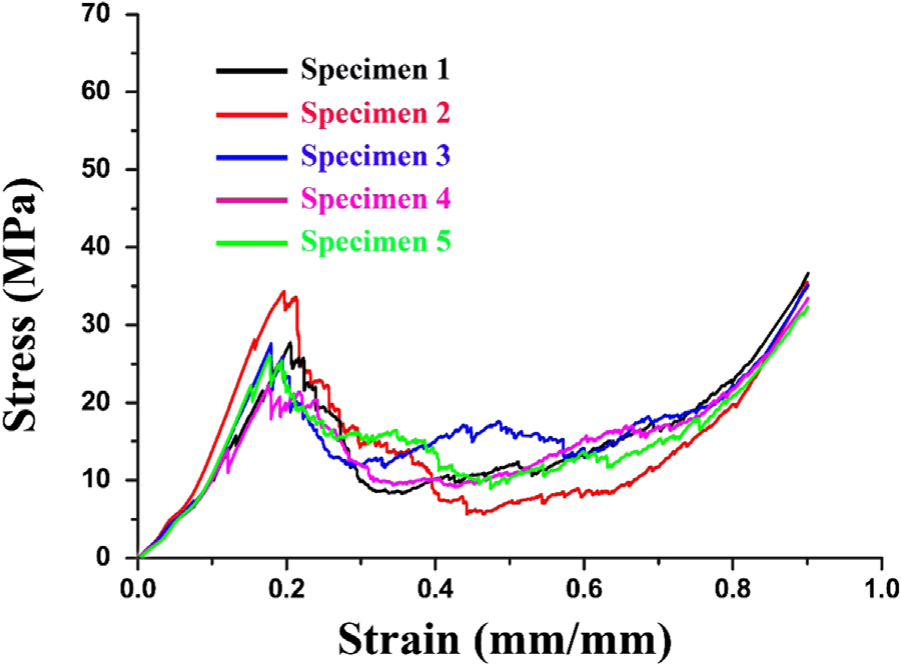
Stress–strain curves of carbon foams.

The compressive stress–strain curves of carbon foams was plotted for each specimen using the force–displacement data. As shown in Fig. 4, the curves of carbon foams with similar density show consistency with each other. When subjected to continuous pressure, the carbon foams exhibit a multi-stage deformation response including a linear elastic region followed by a yield point, a plateau region and graceful failure [6]. Table 1 provides the compressive properties such as the compressive strength, plateau stress and modulus. The compressive strength refers to the peak at the end of the linear elastic region and compressive modulus is defined as the slope of the linear part of the curve [7]. The plateau stress is defined as the average value of the strain at the intercept point of the line with a gradient in the plateau region, while the densification strain was taken as the strain at the point of intersection between the horizontal axis of the plot and the backward extended densification line [8]. The compressive strength increases 54.5% when the bulk density of carbon foams increases from 0.53 g/cm^3^ to 0.59 g/cm^3^. The compressive modulus also increases with increasing density.

## Figures and Tables

**Table 1:** Compressive properties of carbon foams.

**Specimen #**	**Bulk density (g/cm**^**3**^**)**	**Porosity (%)**	**Modulus (MPa)**	**Compressive strength (MPa)**	**Plateau stress (MPa)**	**Densification strain (mm/mm)**
1	0.54	73.0	135.7	27.7	10.7	0.63
2	0.59	70.5	175.1	34.3	6.8	0.70
3	0.54	73.0	155.1	27.6	16.2	0.65
4	0.53	73.5	126.9	22.2	9.9	0.66
5	0.56	72.0	144.9	26.0	10.5	0.66
Avg.	0.56±0.02	72.4±1.1	145.5±16.8	27.6±4.4	10.8±3.4	0.66±0.03

